# Anterior gradient 2 is involved in the post-transcriptional regulation of β-dystroglycan

**DOI:** 10.1080/19768354.2020.1871405

**Published:** 2021-01-17

**Authors:** Eunyoung Lee, Do Hee Lee

**Affiliations:** Department of Bio and Environmental Technology, Seoul Women’s University, Seoul, Korea

**Keywords:** AGR2, β-dystroglycan, actin cytoskeletal network, cell adhesion

## Abstract

Anterior gradient 2 (AGR2) is a protein disulfide isomerase over-expressed in numerous types of cancer. Although AGR2 plays a role in ER homeostasis, its function(s) in tumorigenesis is still elusive. Here we demonstrate that AGR2 is involved in the regulation of the β-subunit of dystroglycan (β-DG), a component of the multi-protein complex linking the extracellular matrix and cytoskeletal network. In breast cancer cells, AGR2 over-expression led to the up-regulation of β-DG but not that of α-DG, while the transcript levels of these subunits were unchanged. Conversely, the reduced expression of AGR2 caused the down-regulation of β-DG. Interestingly, induced expression of AGR2 increased the degree of co-localization of AGR2 and β-DG in the cytoplasm suggesting that AGR2 facilitates the trafficking of β-DG. In addition, AGR2 over-expression caused the re-arrangement of the actin cytoskeletal network. Presumably over-expressed AGR2 up-regulates β-DG post-transcriptionally and facilitates its trafficking, which then causes re-arrangement of the cytoskeletal network, which plays a role in the adhesion and invasion of cancer cells.

## Introduction

AGR2 is considered as a unique member of the protein disulfide isomerase (PDI) superfamily due to the presence of a non-canonical catalytic motif (CXXS) and non-optimal ER retention sequence (KTEL) (Obacz et al. 2015), (Lee and Lee 2017). While further studies are needed to determine if AGR2 functions as a *bona fide* PDI, accumulating evidence indicates that AGR2 plays roles in tumor development by stimulating the proliferation and promoting the metastasis of cancer cells (Lee and Lee 2017). The elevated expression of AGR2, which is observed in many types of malignant cancers, is often utilized as a prognostic marker to evaluate the patient outcomes (Salmans et al. 2013), (Tian et al. 2017). Additionally, AGR2 stabilizes hypoxia inducible factor-1α, indicating a role of this chaperone in the chemoresistance of cancer cells (Li et al. 2015). Furthermore, a number of signaling molecules (e.g. FOXA1 and TGF-β) are shown to regulate the expression of AGR2 in cancer cells (Alsereihi et al. 2019). Despite such findings, the precise role(s) of AGR2 in tumorigenesis is still far from being clearly understood.

Several mechanisms explaining the oncogenic effects of AGR2 have been proposed. Notably it was shown that AGR2 negatively regulates the p53 signaling pathway and thereby confers resistance to DNA damaging stress (Hrstka et al. 2016). AGR2 also promotes the growth and metastasis of prostate cancer cells through the activation of the NF-κB pathway (Jia et al. 1864). In the ER, AGR2 forms a homodimer and modulates the unfolded protein response through its ability to interact with BiP (Grp78) (Ryu et al. 2013). A recent study reported that homo-dimerization of AGR2, which is crucial for the ER proteostasis, is perturbed in disease conditions involving the pro-inflammatory response (Maurel et al. 2019). Although AGR2 is functionally associated with ER homeostasis and several signaling pathways, another line of evidence indicates the role(s) of AGR2 in the regulation of cell adhesion and invasion. Transforming growth factor-beta (TGF-β), a strong promoter of epithelial–mesenchymal transition (EMT), is known to suppress AGR2 expression (Sommerova et al. 2017). EMT is characterized by the loss of epithelial phenotypes including the dissolution of cell–cell junctions, increased deposition of extracellular matrix (ECM) proteins and the re-organization of the actin network (Xu et al. 2009). AGR2 is required for maintaining epithelial characteristics and preventing the activation of factors involved in EMT (Sommerova et al. 2017). In prostate cancer cells, AGR2 promotes cell adhesion by regulating the expression of integrin (Xu et al. 2009). A recent study reported that the effects of AGR2 are mediated by C4.4A, a GPI-linked cell membrane receptor, which requires both laminin (ECM) and integrin (membrane) (Arumugam et al. 2015). Interestingly, an earlier study reported that AGR2 interacts with membrane proteins including C4.4A and dystroglycan (Fletcher et al. 2003). Dystroglycan is a component of the multi-protein complex linking the basement membrane and cytoskeletal network (Barresi and Campbell 2006). These findings raised a possibility that AGR2 affects the adhesion and invasion of cancer cells through the membrane protein dystroglycan.

To investigate their relationship, we studied the effects of AGR2 on dystroglycan. In breast cancer cells, the expression levels of AGR2 protein positively correlated with the protein levels of the β-DG subunit but not that of the α-DG subunit of dystroglycan. By contrast, the gene expression of neither subunit was changed by AGR2. We also found that AGR2 facilitated the trafficking of β-DG and increased the degree of co-localization with β-DG in the cytoplasm, indicating the involvement of AGR2 in the post-transcriptional regulation of β-DG.

## Materials and methods

### Cell culture, plasmids and DNA transfection

MDA-MB-231, MDA-MB-453 and MCF7 breast cancer cells (provided by Prof. C.H. Chung, Seoul National University) were maintained in DMEM containing 10% FBS and 1% penicillin/streptomycin at 37°C and 5% CO_2_. To induce AGR2 expression, cells were plated onto 60-mm dishes (3.0 × 10^5^ cells) overnight and then incubated with 100 μM CoCl_2_ for 24 h. Expression constructs (in pcDNA 3.1(+) Zeo vector) for AGR2 were described previously (Ryu et al. 2013). Lentiviral shRNA plasmids for knock-down of AGR2 (in pLKO.1 vector) were purchased from Dharmacon™ (GE Healthcare). Transient transfection of the plasmids was carried out by using jetPEI® (Polyplus-Transfection). After 24˗72 h of transfection, cells were harvested and processed for further analyses.

### Immunoblot analysis and antibodies

After transfection, cells were harvested and lysed in NP-40 lysis buffer (20 mM Tris-HCl, pH 7.5; 150 mM NaCl; and 1% NP-40) supplemented with protease inhibitors (Roche). The clarified cell lysate (usually 50 μg protein) was applied onto 10% SDS-PAGE gels and then transferred to PVDF membranes. The membranes were probed with the primary antibodies followed by incubation with HRP-conjugated secondary antibodies. The proteins were visualized by ECL reagents (GE Healthcare). Monoclonal antibodies used in this study were anti-AGR2 antibody (1:1,000; abcam), anti-α-DG antibody (1:1,000; Cell Signaling), anti-β-DG antibody (1:1,000; Santa Cruz), MANDAG for β-DG (1:200; DSHB, University of Iowa), anti-MMP2 antibody (1:1,000; abcam), anti-MMP9 antibody (1:1,000; Cell Signaling), and anti-β-actin antibody (1:20,000; abcam).

### Immunofluorescence microscopy

Cells seeded onto coverslips were grown to 30˗40% confluence and then transfected with plasmids or treated with CoCl_2_ as described above. After 24˗72 h, cells were fixed with 3.7% formaldehyde for 15 min and permeabilized with 0.5% Triton X-100 for 15 min at room temperature. After blocking with 10% normal goat serum, coverslips were incubated with primary antibodies (1:200) or Texas Red™-phalloidin (for F-actin staining; Thermo Fisher) at 4°C overnight. After washing, coverslips were incubated with secondary antibodies conjugated with FITC or Cy3 (1:200; Bethyl) for 1 h at room temperature. Coverslips were incubated with mounting solution containing DAPI and observed under a fluorescence microscope (Zeiss).

### Quantitative RT–PCR analysis

Total RNA was extracted using the Trizol reagent (Invitrogen), and cDNA was synthesized from 1 μg of total RNA using the PrimerScript RT reagent kit (Takara). Quantitative PCR analysis was carried out using the SYBR green mix (Enzo). All samples were normalized to GAPDH and were calculated using ΔΔCt method for mRNA quantification. Sequences of primers used in this study are listed in Table 1.

**Table 1.** Primer Sequences for Semi-quantitative RT-PCR Analysis.

Gene	Primer Sequence
AGR2	Forward: 5´-ACAAAGGACTCTCGACCCAAA-3´ Reverse: 5´-GTGGGCACTCATCCAAGTGA-3´
α-DG	Forward: 5´-CTTCAACAGCAACAGCCAGCTCAT-3´ Reverse: 5´-TGGTGCTACAGTTTCGGTCTCCAA-3´
β-DG	Forward: 5´-GCCTGACTTTAAGGCCACAAGCAT-3´ Reverse: 5´-CAATGATGCCAGCAATGAGCAGGA-3´
MMP9	Forward: 5´-GCACTGCAGGATGTCATAGG-3´ Reverse: 5´-ACGACGTCTTCCAGTACCGA-3´
GAPDH	Forward: 5´-AGCAATGCCTCCTGCACCACCAAC-3´ Reverse: 5´-CCGGAGGGGCCATCCACAGTC-3´

### Statistical analysis

The results are expressed as mean ± SEM from triplicate experiments. Mean values were analyzed by Student t-test, and *p* <0.05 was considered statistically significant.

## Results

Previously dystroglycan and C4.4A (LYPD3) were identified as AGR2 binding partners (Fletcher et al. 2003). While it was later shown that AGR2 directly binds to C4.4A, the evidence for the interaction between AGR2 and dystroglycan has never been validated (Arumugam et al. 2015). Another study reported that the abrogation of AGR2 reduced the attachment of prostate cancer cells to ECM proteins including fibronectin and laminin (Chanda et al. 2014). These results implicated that AGR2 possibly functions in the regulation of the laminin-receptor dystroglycan and thereby affects ECM remodeling. Here, we focused on the transmembrane β-DG subunit, because it is associated with the actin filament network and intracellular signaling cascade, whereas the extracellular α-DG subunit mainly interacts with a variety of ECM proteins (Moore and Winder 2010).

**Figure 1.**
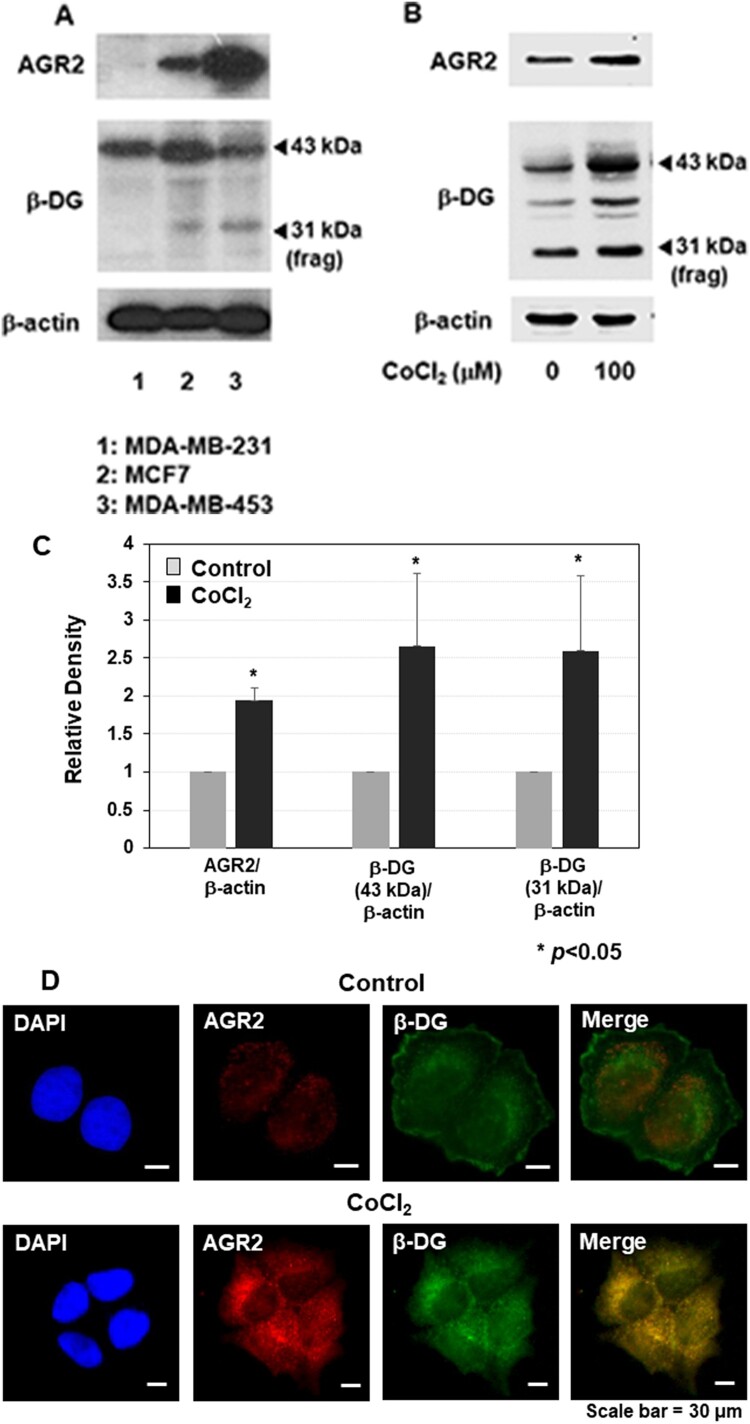
Increased expression of AGR2 correlates with the up-regulation of β-DG. (A) Three representative breast cancer cell lines with differential AGR2 expression levels (MDA-MB-231, MCF7 and MDA-MB-453) were chosen, and the protein levels of AGR2 and β-DG were compared (B) To induce AGR2 expression, MCF7 cells were treated with 100 μM CoCl_2_, and the protein levels of AGR2 and β-DG were measured (C) For quantification of the western-blot results in Fig 1B, the images were scanned and the densitometer analysis of corresponding bands were carried out (error bars; SEM from triplicates, *p*<0.05). (D) Immunofluorescence microscopic analysis of AGR2 (Cy3) and β-DG (FITC) in MCF7 cells. Nuclei were visualized with DAPI staining (magnification, 400X)

First, we selected three representative breast cancer cell lines with differential AGR2 expression (MDA-MB-231, MCF7, and MDA-MB-453 cells) and compared the protein levels of β-DG. In breast cancer cells showing robust expression of AGR2 (i.e. MCF7 and MDA-MB-453), β-DG subunit was clearly up-regulated, whereas it was unchanged in cells expressing little or no AGR2 (i.e. MDA-MB-231) (Figure 1(A)). To confirm this result, we treated MCF7 cells with 100 μM CoCl_2_, a hypoxia mimetic agent known to induce AGR2 (Hing et al. 2010), and examined its effects on the protein levels of AGR2 and β-DG. As expected, CoCl_2_ treatment increased the levels of both AGR2 and β-DG (Figure 1(B)). Quantitation of β-DG proteins (both the full-length 43 kDa protein and 31 kDa fragment) also revealed a positive correlation between AGR2 and β-DG (Figure 1(C)). Interestingly, immunofluorescence microscopic analysis showed that β-DG, which was largely membrane bound and did not overlap with AGR2 in the untreated cells, was translocated into the cytoplasm and showed a high degree of co-localization with AGR2 in CoCl_2_-treated MCF7 cells (Figure 1(D)).

**Figure 2.**
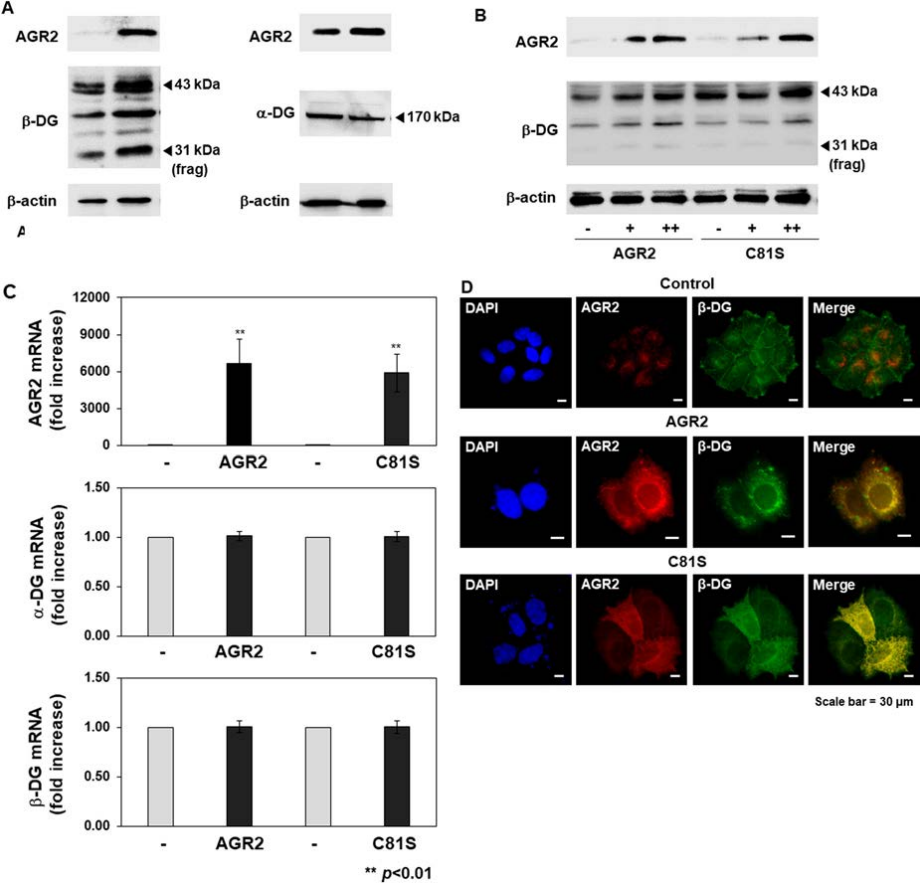
Over-expression of AGR2 increases β-DG protein levels and co-localization of AGR2 and β-DG inside cells. (A) Expression vector for AGR2 was transfected into MCF7 cells and the protein levels of AGR2, β-DG, and α-DG were measured. (B) Effects of wild-type AGR2 and C81S mutant of AGR2 on β-DG protein levels were compared (+; 1 μg, ++; 2 μg plasmid DNA transfected). (C) Effects of AGR2 over-expression (normal and C81S mutant) on α-DG and β-DG mRNA levels were examined. (D) Immunofluorescence microscopic analysis of AGR2 (Cy3) and β-DG (FITC) in AGR2-over-expressing MCF7 cells. Nuclei were visualized with DAPI staining (magnification, 400×).

**Figure 3.**
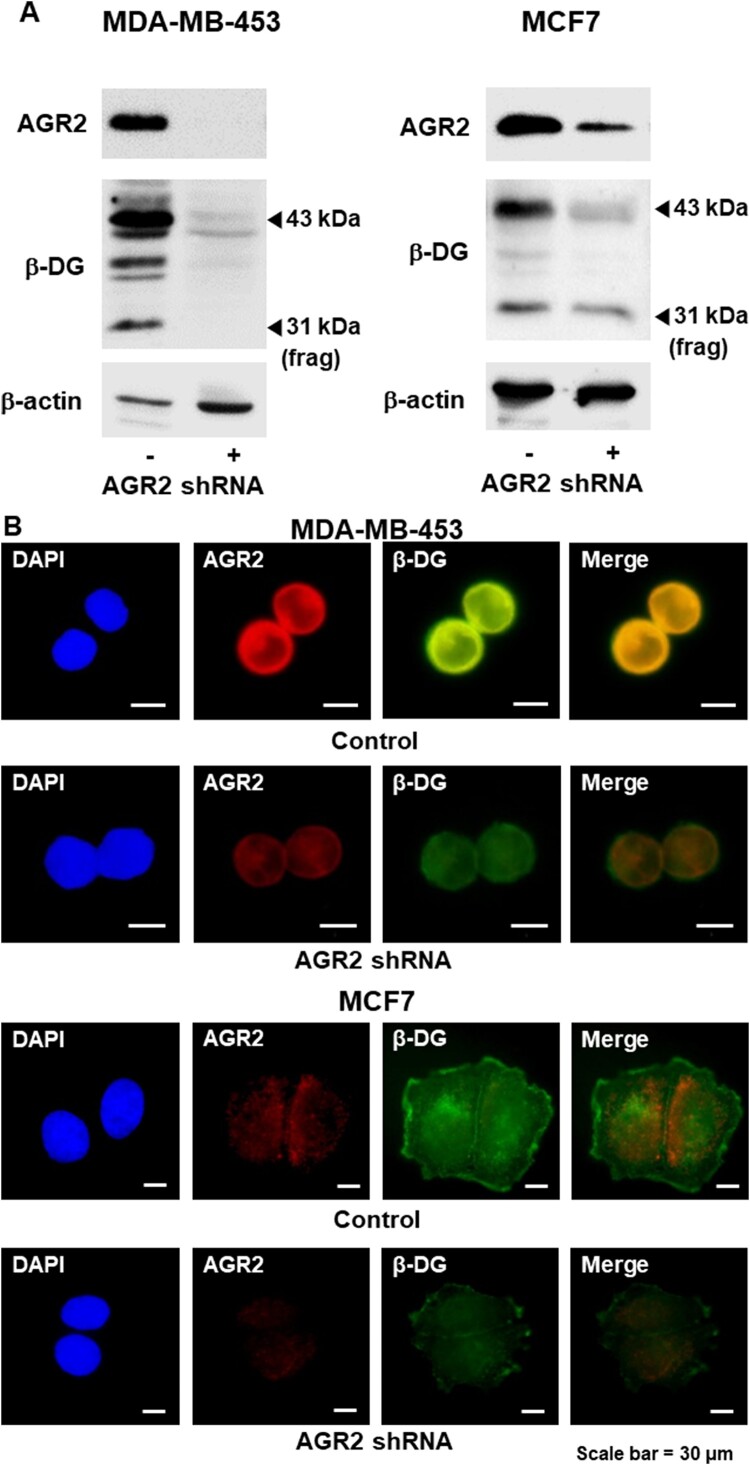
Reduced expression of AGR2 leads to the down-regulation of β-DG. (A) AGR2 shRNA vector was transfected into MDA-MB-453 cells and MCF7 cells and then the protein levels of AGR2 and β-DG were examined. (B) Expression and sub-cellular localization of AGR2 (Cy3) and β-DG (FITC) in AGR2-silenced MDA-MB-453 cells (upper panel) and MCF7 cells (lower panel) were analyzed by immunofluorescence microscopy. Nuclei were visualized with DAPI staining (magnification, 400×).

To verify these results, we transfected the AGR2 expression vector into MCF7 cells and compared the protein levels of AGR2 and β-DG. Similar to the effects of CoCl_2_, over-expression of AGR2 caused up-regulation of the β-DG protein in MCF7 cells. In contrast to β-DG, the protein level of the extracellular α-DG subunit did not change in AGR2 over-expressing cells indicating that the effect of AGR2 is specific for β-DG (Figure 2(A)). To determine if the catalytic activity of AGR2 is crucial for up-regulation of β-DG, we compared the effects of normal AGR2 and the catalytic mutant (C81S) of AGR2 on β-DG. As shown in Figure 2(B), AGR2 did not require its PDI activity to increase the level of β-DG (although catalytically inactive C81S AGR2 was slightly less effective than the normal AGR2). These results implicate that the chaperone function of AGR2 rather than the disulfide bond-forming activity is important for up-regulation of β-DG. To test if the up-regulation of β-DG is due to the increased transcription, we measured the mRNA levels of both subunits. Quantitative RT–PCR analysis showed that induced expression of AGR2 (either normal AGR2 or C81S mutant) did not change the transcript-level expression of these subunits indicating that AGR2 exerts its effects on dystroglycan post-transcriptionally (Figure 2(C)). Immunofluorescence analysis confirmed that over-expression of AGR2 not only up-regulated β-DG but also increased the degree of cytoplasmic co-localization of β-DG and AGR2, suggesting that AGR2 facilitates the trafficking of transmembrane β-DG (Figure 2(D)). For the knock-down experiments, we used MDA-MB-453 and MCF7 cells showing the robust expression of AGR2. As expected, AGR2 knock-down greatly reduced the levels of β-DG protein in both MDA-MB-453 and MCF7 cells (Figure 3(A)). Immunofluorescence analysis showed that the reduced expression of AGR2 effectively down-regulated β-DG protein in the breast cancer cells (AGR2 knock-down did not change co-localization) (Figure 3(B)).

**Figure 4.**
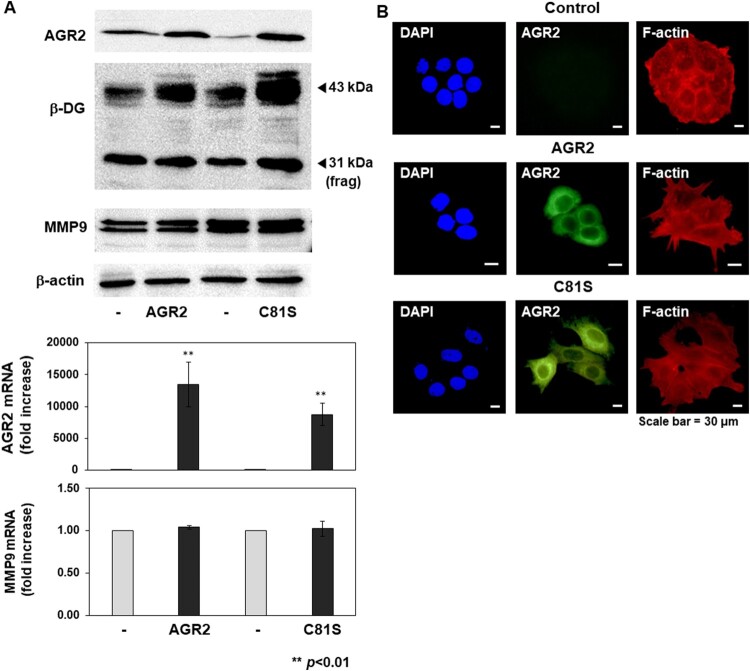
Effects of AGR2 over-expression on MMP-9 levels and F-actin arrangement. (A) Expression vectors for AGR2 (normal and C81S mutant) were transfected into MCF7 cells, and the protein levels of AGR2, β-DG, and MMP-9 were examined (*upper panel*). Transcript-level expression of AGR2 and MMP9 was also measured and compared (error bars; SEM from triplicates, *p*<0.01) (*lower panel*). (B) Effects of AGR2 over-expression on the arrangement of F-actin in MCF7 cells. Expression and sub-cellular localization of AGR2 (FITC) and F-actin (Texas Red-phalloidin) were analyzed by immunofluorescence microscopy. Nuclei were visualized with DAPI staining (magnification, 400×).

Accumulation of the fragment of β-DG has been frequently observed in human breast cancer cell lines, underlying the importance of β-DG proteolysis in tumorigenesis (Sgambato and Brancaccio 2005). Several proteolytic enzymes, including MMP-2, −9 and γ-secretase, are linked to β-DG processing. In fact, it was demonstrated that β-DG is a physiological target of MMP-9 (Michaluk et al. 2007). To determine if the effects of AGR2 on β-DG are due to the increased expression of MMP-9, we measured gene expression as well as the level of MMP-9 protein in AGR2 over-expressing MCF7 cells. As shown in Figure 4(A), AGR2 over-expression changed neither gene expression nor protein level of MMP-9, ruling out a possibility that the effects of AGR2 on β-DG is mediated by MMP-9-mediated increased proteolysis of β-DG. We also observed that AGR2 did not change the level of MMP-2 protein either (data not shown).

Dystroglycan is a molecule linking the ECM and the cytoskeletal network and plays an important role in tumor development. The cytoplasmic domain of β-DG associates with a variety of cytoskeletal components, including actin, dystrophin, as well as the proteins involved in signal transduction (Moore and Winder 2010), (Sgambato and Brancaccio 2005). In lung cancer cells, AGR2 knock-down led to the re-organization of F-actin and increased the formation of stress fibers (Sommerova et al. 2017). We hypothesized that AGR2 affects the organization of the actin cytoskeletal network through the regulation of β-DG, which associates with the components of the actin cytoskeletal network and functions as a scaffold for the signaling cascade. To test this possibility, we examined if the arrangement of cytoskeletal F-actin is altered by AGR2 over-expression. As expected, over-expression of AGR2 in MCF7 cells caused the re-arrangement of cytoskeletal F-actin protein and substantially reduced the formation of actin stress fibers in MCF7 cells (Figure 4(B)). These findings together implicate a regulatory role of AGR2 and β-DG in the organization of intracellular actin cytoskeletal network in cancer cells.

## Discussion

Although the model underlying the role(s) of AGR2 in the formation and/or re-arrangement of disulfide bonds in client proteins is appealing, it is unclear if the PDI activity is responsible for all the effects attributed to AGR2. On the contrary, the presence of AGR2 in the extracellular milieu, especially in cancer cells, implies that AGR2 exerts pro-oncogenic effects via diverse mechanism(s) (Chevet et al. 2013). In a number of cancer cell models, AGR alters cell adhesion suggesting that AGR2 carries out ER-independent function(s) (Chanda et al. 2014), (Chevet et al. 2013). A recent study provided evidence that extracellular AGR2 interacts with ECM proteins, disrupts cell–cell adhesion and promotes the formation of invasive structure (Fessart et al. 2016). These findings indicate the importance of interaction between AGR2 and ECM proteins and/or the membrane proteins. An earlier report employing yeast two-hybrid analysis suggested C4.4A (LYPD3) and dystroglycan as the membrane receptors of AGR2 (Chanda et al. 2014). A recent study identifying the proteins interacting with AGR2 showed that over 40% of candidates are membrane associated and that the oncogenic membrane receptor EpCAM is one such candidate (Mohtar et al. 2018). It was later demonstrated that C4.4A directly associates with AGR2, and a positive correlation exists between the expression of AGR2 and C4.4A in pancreatic cancer tissues (Arumugam et al. 2015). By contrast, the protein–protein interaction between dystroglycan and AGR2 has never been validated, and it was even suggested that AGR2 does not bind to dystroglycan (Arumugam et al. 2015). Despite such reports, dystroglycan is well-known for its role in cell adhesion and trafficking of ECM proteins (Moore and Winder 2010), (Leonoudakis et al. 2014) raising a possibility that dystroglycan is involved in AGR2-mediated cell adhesion and metastasis.

Initially, we utilized database available in GOBO site (Gene expression-based Outcome for Breast cancer Online; http://co.bmc.lu.se/gobo/gobo.pl) to compare the expression level of AGR2 and dystroglycan in more than 1,800 samples of breast tumors. AGR2, strongly associated with ER-positive breast cancer, is up-regulated in luminal A and B subtypes as well as HER2-positive breast cancer. On the contrary, dystroglycan is not associated with ER-positive breast cancer nor specifically up-regulated in certain subtypes of breast cancer (data not shown). We also examined RNA-Seq data of human breast cancer cell lines (provided by Dr. Joe Gray, OHSU) and found no positive correlation in transcript-level expression between AGR2 and dystroglycan (data not shown). In pancreatic cancer cells, AGR2 up-regulates the downstream effectors (e.g. cathepsin B and D) without changing their mRNA levels supporting that AGR2 could exert its effects on client proteins post-transcriptionally (Dumartin et al. 2011). We also showed that AGR2 similarly up-regulates the β-DG protein without affecting its transcript-level. Although it was believed that PDI activity is important, we found that the catalytic activity of AGR2 is not required for up-regulation of β-DG. Similar to our results, the interaction between AGR2 and the proteins involved in cell adhesion process does not require the functional thioredoxin-like domain (Fessart et al. 2016). These observations suggested that AGR2 does not require PDI activity but instead the interaction between AGR2 and cell surface receptors plays a role in the adhesion and metastasis of cancer cells. Although yeast two-hybrid analysis showed that AGR2 binds to α-DG, no evidence for their *in vivo* interaction has been provided. We did not observe the interaction between AGR2 and β-DG by co-immunoprecipitation either (data not shown). However, the results showing the co-localization of AGR2 and β-DG suggested that over-expression of AGR2 may promote the trafficking of membrane bound β-DG into the cytoplasm, in which more stable association of these proteins may take place. Interestingly, the interaction analysis of AGR2 (cytosolic) and EpCAM (membrane bound) also showed a similar pattern, and these proteins formed a stable complex inside cells (Mohtar et al. 2018).

Dystroglycan is translated from a single mRNA transcript and then post-translationally cleaved into extracellular α-DG and transmembrane β-DG subunits (Barresi and Campbell 2006). We focused on the β-DG because this subunit binds to proteins involved in the cytoskeletal network as well as proteins implicated in intracellular signal transduction (Brennan et al. 2004). In platelets, β-DG acts as an interplay protein between actin and microtubules and also binds to focal adhesion complex components (Cerecedo et al. 2007). Our result demonstrate that AGR2 causes the re-arrangement of actin stress fibers, highlighting that the effects of AGR2 on the adhesion of cancer cells is mediated (at least partially) via the role of β-DG as a platform for both the cytoskeletal network and focal adhesion complex. AGR2 promotes the migration of keratinocytes through the activation of FAK and JNK pathways (Zhu et al. 2017). It would be interesting to test if β-DG affects the activation of such signaling pathways in cells over-expressing AGR2. It was recently reported that β-DG undergoes retrograde intracellular trafficking from the plasma membrane to the nucleus via the endosomes-ER network (Tian et al. 2017). Furthermore, the accumulation of β-DG in the nucleus led to alteration in the nuclear architecture (Jia et al. 1864). In this regard, the dysregulation of intracellular trafficking of β-DG may diminish the nuclear content of this subunit. Moreover, accumulation of the intracellular domain of β-DG (the proteolytic fragment) in the nucleolus changed ribosome profiling and suppressed rRNA expression (Azuara-Medina et al. 2019).

Even though we demonstrated that AGR2 exerts its effects post-transcriptionally, we are yet to elucidate the mechanism(s) by which this ER chaperone regulates β-DG. It was reported that induced nuclear accumulation of β-DG promotes its degradation by the ubiquitin-proteasome system (UPS), indicating that cells tightly control the nuclear content of β-DG to maintain proper nuclear activity (Jia et al. 1864). It is thus conceivable that the over-expression of AGR2 leads to the stabilization of β-DG and thereby causes its up-regulation within cells. The observations that the formation of the 31 kDa fragment of β-DG was not always proportional to the extent of AGR2 expression also suggest that AGR2 perhaps up-regulates β-DG by inhibiting its degradation via UPS rather than by promoting the proteolytic cleavage via MMPs. Alternatively it is possible that another yet-to-be identified proteolytic enzyme(s) mediates the proteolysis of β-DG since it was shown that β-DG is cleaved by MMP-14 in skeletal muscle lacking MMP-2 and −9 (Fukai et al. 2017).

Here, we present evidence that AGR2 regulates the β-subunit of a cell adhesion receptor dystroglycan post-transcriptionally. Although the mechanism(s) by which AGR2 up-regulates β-DG and the up-regulated β-DG influence the adhesion and invasiveness of cancer cells need to be elucidated, our findings indicate that the two proteins seemingly involved in different cellular pathways function together in cell migration/invasion and thereby promote the development and metastasis of cancer cells.
